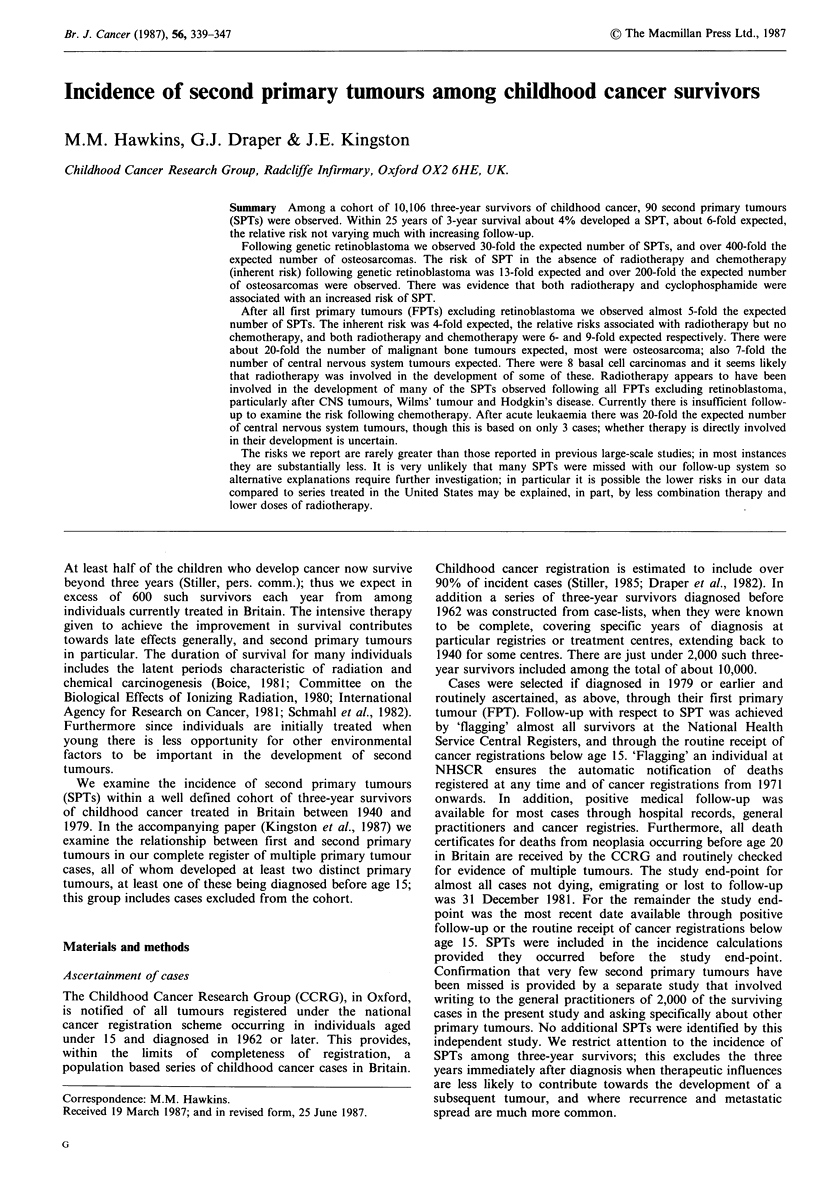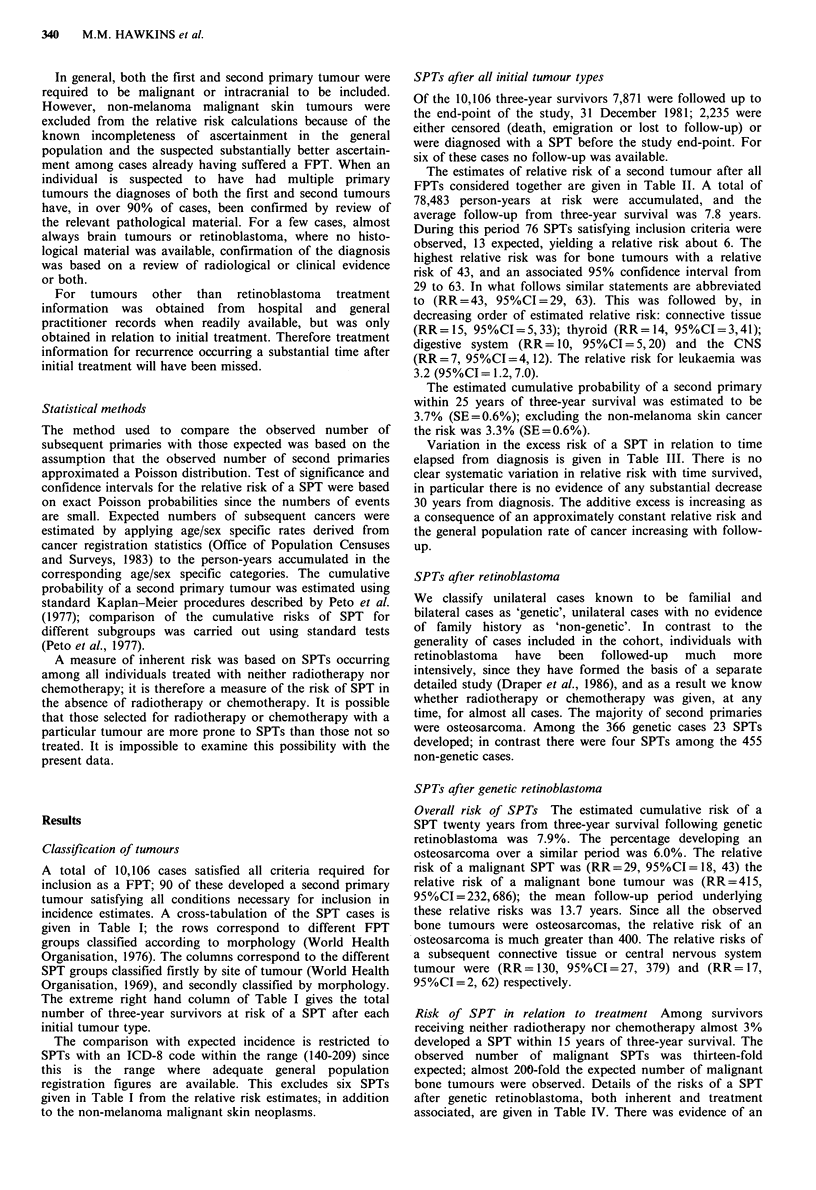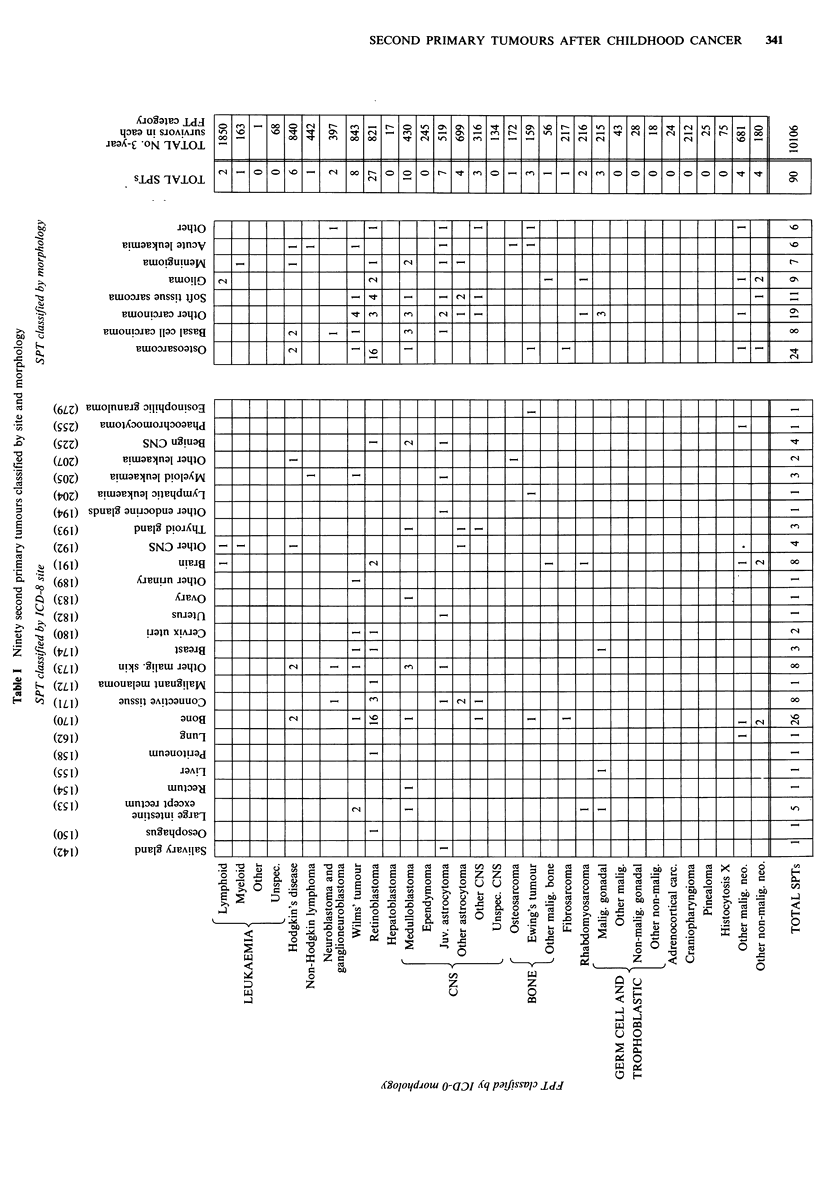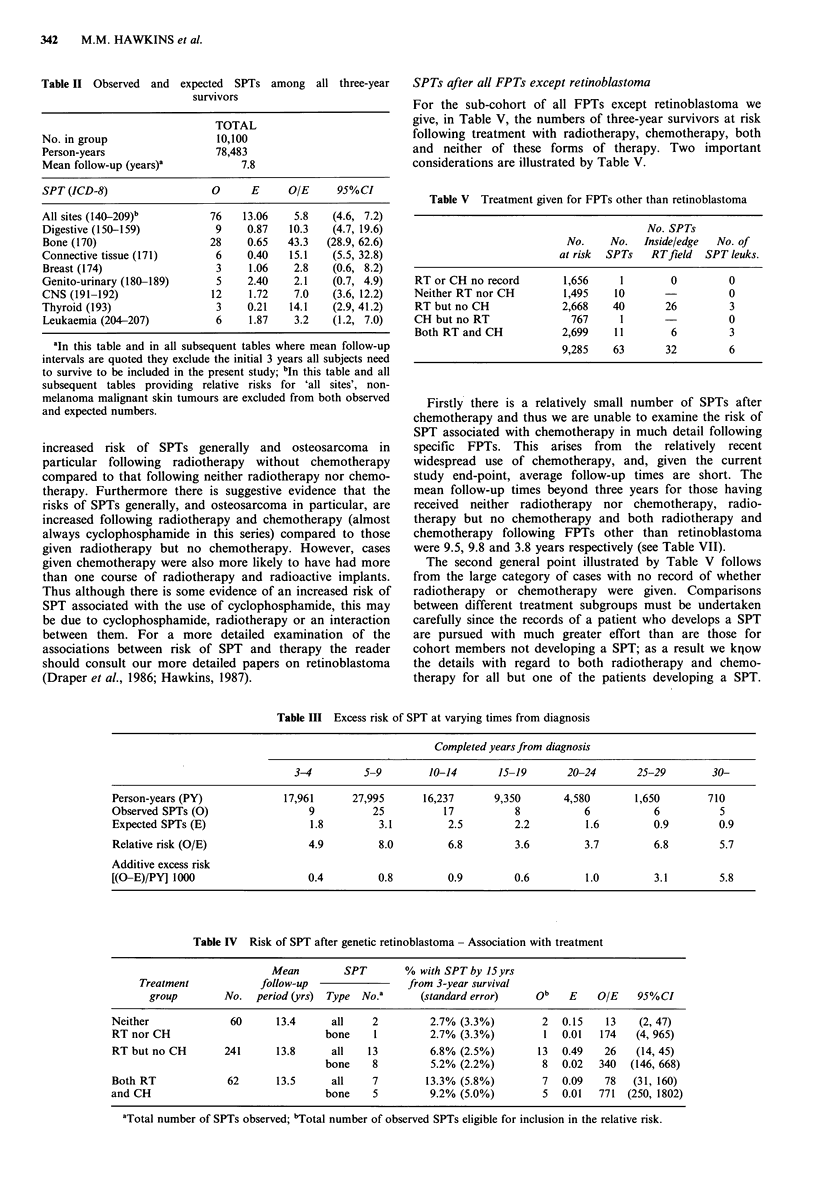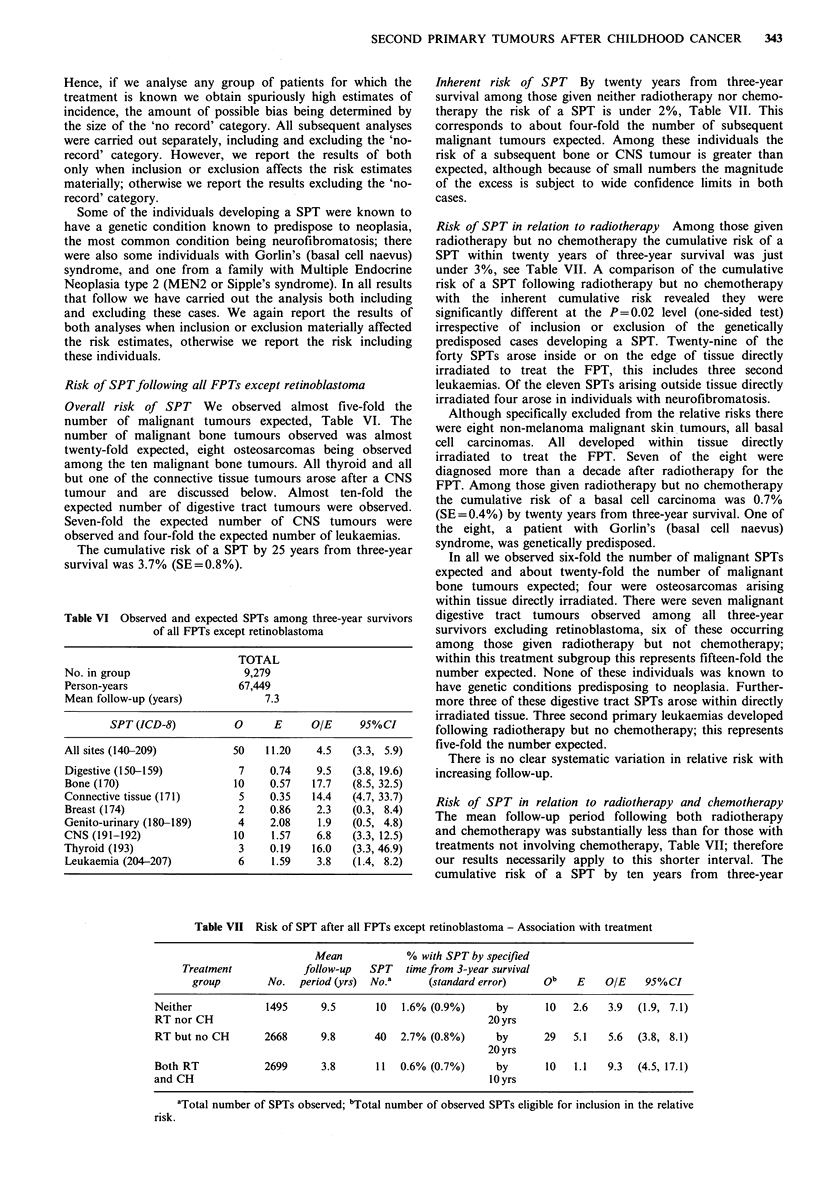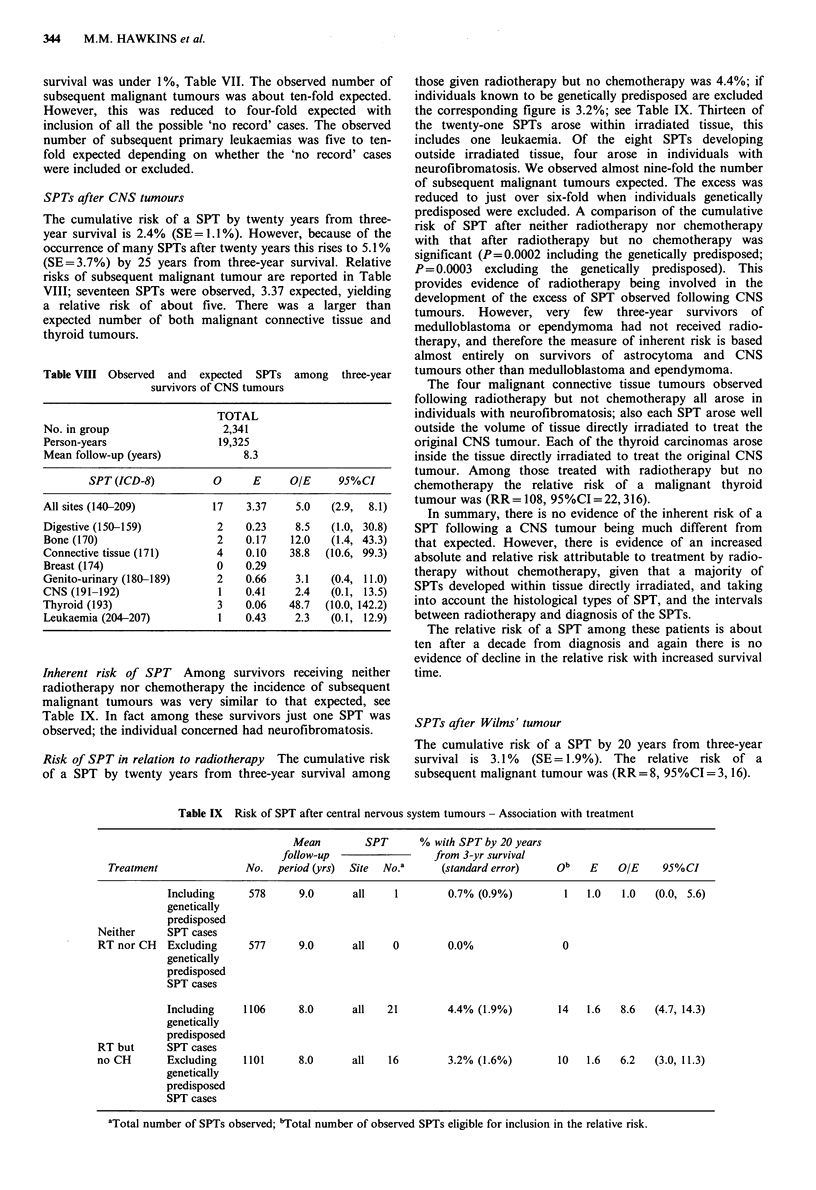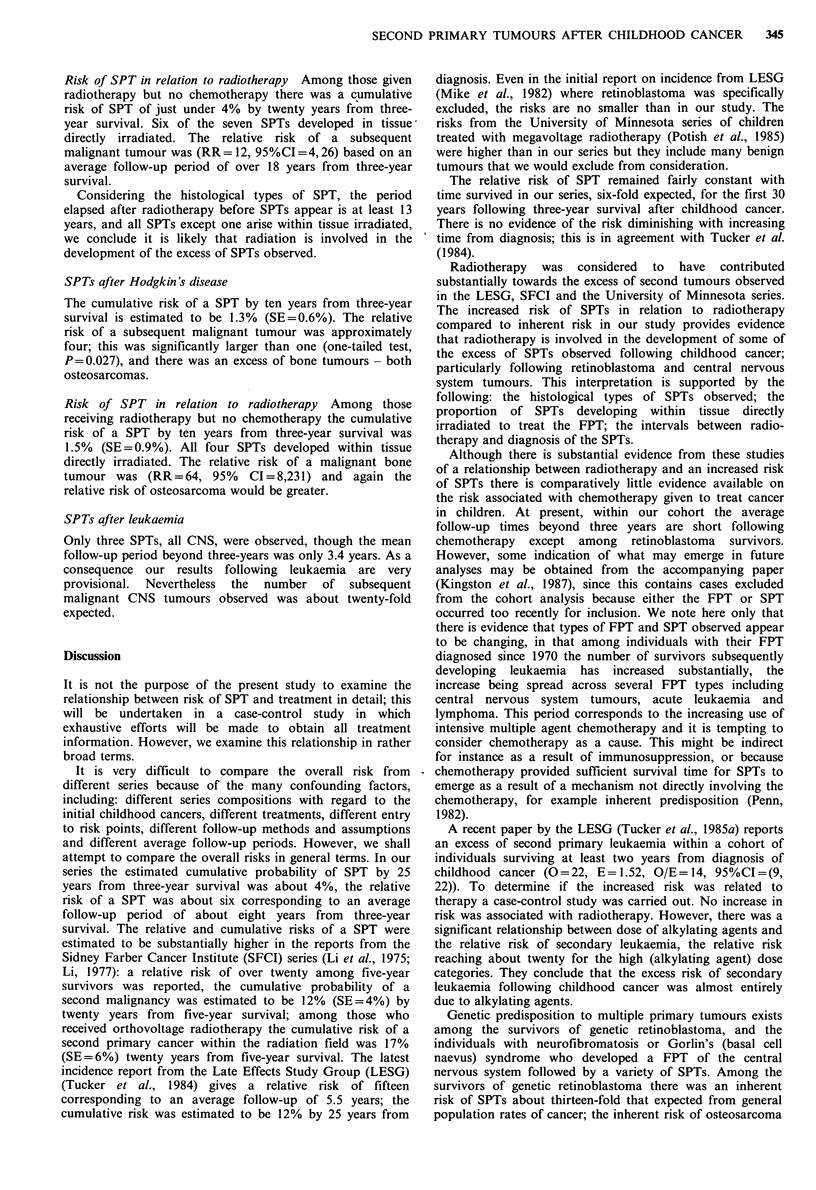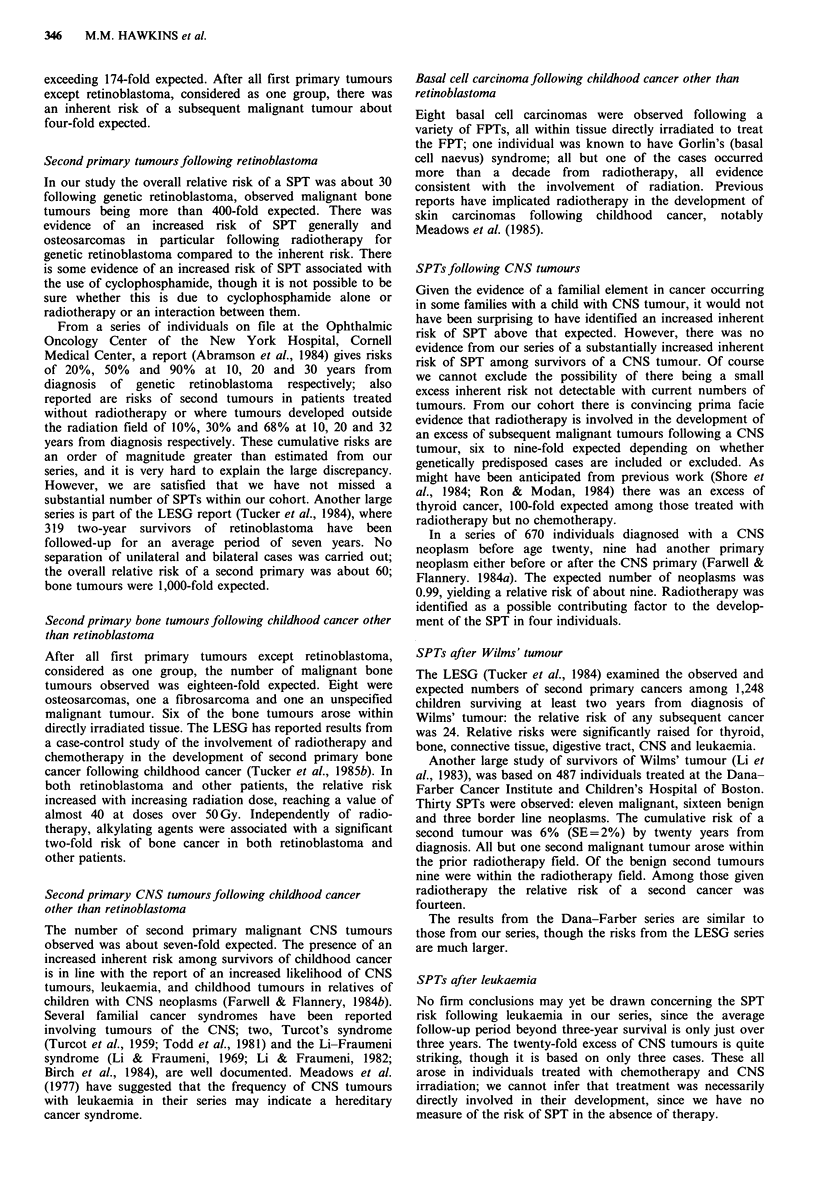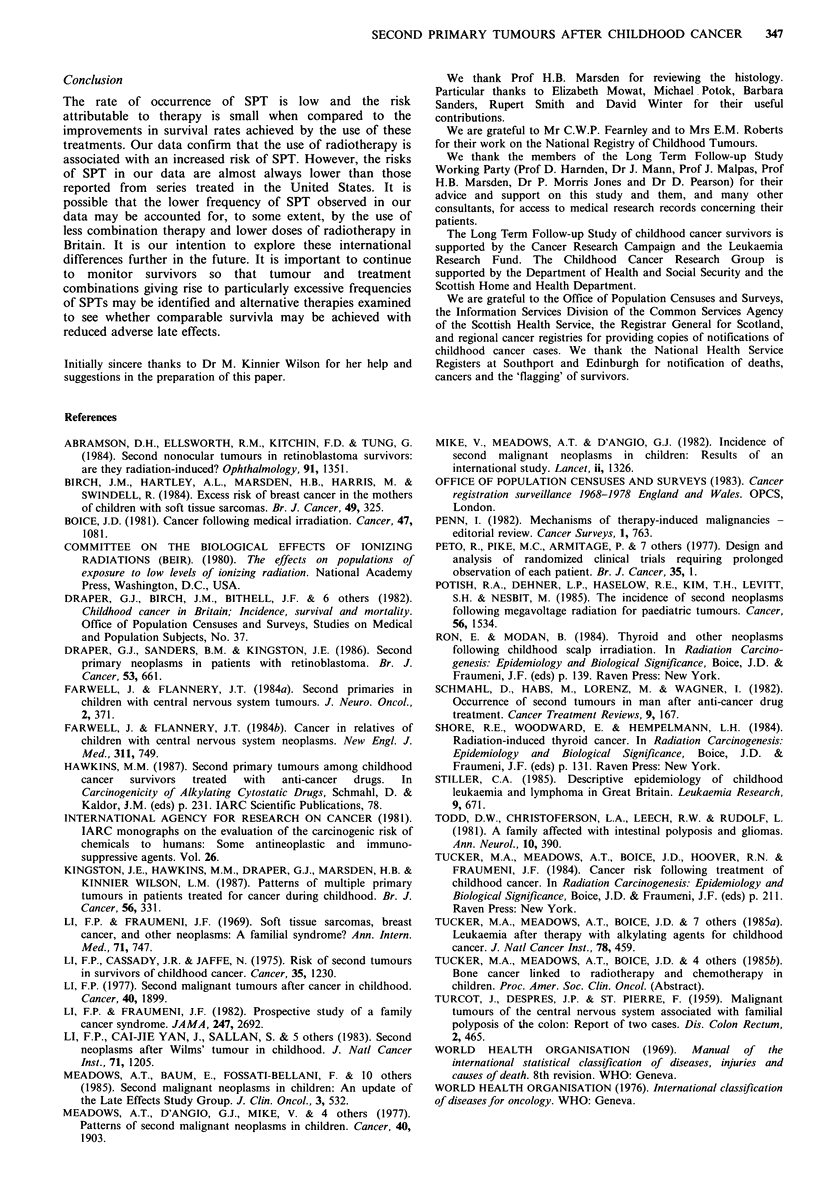# Incidence of second primary tumours among childhood cancer survivors.

**DOI:** 10.1038/bjc.1987.200

**Published:** 1987-09

**Authors:** M. M. Hawkins, G. J. Draper, J. E. Kingston

**Affiliations:** Childhood Cancer Research Group, Radcliffe Infirmary, Oxford, UK.

## Abstract

Among a cohort of 10,106 three-year survivors of childhood cancer, 90 second primary tumours (SPTs) were observed. Within 25 years of 3-year survival about 4% developed a SPT, about 6-fold expected, the relative risk not varying much with increasing follow-up. Following genetic retinoblastoma we observed 30-fold the expected number of SPTs, and over 400-fold the expected number of osteosarcomas. The risk of SPT in the absence of radiotherapy and chemotherapy (inherent risk) following genetic retinoblastoma was 13-fold expected and over 200-fold the expected number of osteosarcomas were observed. There was evidence that both radiotherapy and cyclophosphamide were associated with an increased risk of SPT. After all first primary tumours (FPTs) excluding retinoblastoma we observed almost 5-fold the expected number of SPTs. The inherent risk was 4-fold expected, the relative risks associated with radiotherapy but no chemotherapy, and both radiotherapy and chemotherapy were 6- and 9-fold expected respectively. There were about 20-fold the number of malignant bone tumours expected, most were osteosarcoma; also 7-fold the number of central nervous system tumours expected. There were 8 basal cell carcinomas and it seems likely that radiotherapy was involved in the development of some of these. Radiotherapy appears to have been involved in the development of many of the SPTs observed following all FPTs excluding retinoblastoma, particularly after CNS tumours, Wilms' tumour and Hodgkin's disease. Currently there is insufficient follow-up to examine the risk following chemotherapy. After acute leukaemia there was 20-fold the expected number of central nervous system tumours, though this is based on only 3 cases; whether therapy is directly involved in their development is uncertain. The risks we report are rarely greater than those reported in previous large-scale studies; in most instances they are substantially less. It is very unlikely that many SPTs were missed with our follow-up system so alternative explanations require further investigation; in particular it is possible the lower risks in our data compared to series treated in the United States may be explained, in part, by less combination therapy and lower doses of radiotherapy.


### Incidence of second primary tumours among childhood cancer survivors

M.M. Hawkins, G.J. Draper & J.E. Kingston

*Childhood Cancer Research Group, Radcliffe Infirmary, Oxford OX2 6HE, UK.*

**Summary** Among a cohort of 10,106 three-year survivors of childhood cancer, 90 second primary tumours (SPTs) were observed. Within 25 years of 3-year survival about 4% developed a SPT, about 6-fold expected, the relative risk not varying much with increasing follow-up.

Following genetic retinoblastoma we observed 30-fold the expected number of SPTs, and over 400-fold the expected number of osteosarcomas. The risk of SPT in the absence of radiotherapy and chemotherapy (inherent risk) following genetic retinoblastoma was 13-fold expected and over 200-fold the expected number of osteosarcomas were observed. There was evidence that both radiotherapy and cyclophosphamide were associated with an increased risk of SPT.

After all first primary tumours (FPTs) excluding retinoblastoma we observed almost 5-fold the expected number of SPTs. The inherent risk was 4-fold expected, the relative risks associated with radiotherapy but no chemotherapy, and both radiotherapy and chemotherapy were 6- and 9-fold expected respectively. There were about 20-fold the number of malignant bone tumours expected, most were osteosarcoma; also 7-fold the number of central nervous system tumours expected. There were 8 basal cell carcinomas and it seems likely that radiotherapy was involved in the development of some of these. Radiotherapy appears to have been involved in the development of many of the SPTs observed following all FPTs excluding retinoblastoma, particularly after CNS tumours, Wilms' tumour and Hodgkin's disease. Currently there is insufficient follow-up to examine the risk following chemotherapy. After acute leukaemia there was 20-fold the expected number of central nervous system tumours, though this is based on only 3 cases; whether therapy is directly involved in their development is uncertain.

The risks we report are rarely greater than those reported in previous large-scale studies; in most instances they are substantially less. It is very unlikely that many SPTs were missed with our follow-up system so alternative explanations require further investigation; in particular it is possible the lower risks in our data compared to series treated in the United States may be explained, in part, by less combination therapy and lower doses of radiotherapy.

At least half of the children who develop cancer now survive beyond three years (Stiller, pers. comm.); thus we expect in excess of 600 such survivors each year from among individuals currently treated in Britain. The intensive therapy given to achieve the improvement in survival contributes towards late effects generally, and second primary tumours in particular. The duration of survival for many individuals includes the latent periods characteristic of radiation and chemical carcinogenesis (Boice, 1981; Committee on the Biological Effects of Ionizing Radiation, 1980; International Agency for Research on Cancer, 1981; Schmahl *et al.*, 1982). Furthermore since individuals are initially treated when young there is less opportunity for other environmental factors to be important in the development of second tumours.

We examine the incidence of second primary tumours (SPTs) within a well defined cohort of three-year survivors of childhood cancer treated in Britain between 1940 and 1979. In the accompanying paper (Kingston *et al.*, 1987) we examine the relationship between first and second primary tumours in our complete register of multiple primary tumour cases, all of whom developed at least two distinct primary tumours, at least one of these being diagnosed before age 15; this group includes cases excluded from the cohort.

### Materials and methods

### *Ascertainment of cases*

The Childhood Cancer Research Group (CCRG), in Oxford, is notified of all tumours registered under the national cancer registration scheme occurring in individuals aged under 15 and diagnosed in 1962 or later. This provides, within the limits of completeness of registration, a population based series of childhood cancer cases in Britain. Childhood cancer registration is estimated to include over 90% of incident cases (Stiller, 1985; Draper *et al.*, 1982). In addition a series of three-year survivors diagnosed before 1962 was constructed from case-lists, when they were known to be complete, covering specific years of diagnosis at particular registries or treatment centres, extending back to 1940 for some centres. There are just under 2,000 such three-year survivors included among the total of about 10,000.

Cases were selected if diagnosed in 1979 or earlier and routinely ascertained, as above, through their first primary tumour (FPT). Follow-up with respect to SPT was achieved by 'flagging' almost all survivors at the National Health Service Central Registers, and through the routine receipt of cancer registrations below age 15. 'Flagging' an individual at NHSCR ensures the automatic notification of deaths registered at any time and of cancer registrations from 1971 onwards. In addition, positive medical follow-up was available for most cases through hospital records, general practitioners and cancer registries. Furthermore, all death certificates for deaths from neoplasia occurring before age 20 in Britain are received by the CCRG and routinely checked for evidence of multiple tumours. The study end-point for almost all cases not dying, emigrating or lost to follow-up was 31 December 1981. For the remainder the study end-point was the most recent date available through positive follow-up or the routine receipt of cancer registrations below age 15. SPTs were included in the incidence calculations provided they occurred before the study end-point. Confirmation that very few second primary tumours have been missed is provided by a separate study that involved writing to the general practitioners of 2,000 of the surviving cases in the present study and asking specifically about other primary tumours. No additional SPTs were identified by this independent study. We restrict attention to the incidence of SPTs among three-year survivors; this excludes the three years immediately after diagnosis when therapeutic influences are less likely to contribute towards the development of a subsequent tumour, and where recurrence and metastatic spread are much more common.

Correspondence: M.M. Hawkins.
Received 19 March 1987; and in revised form, 25 June 1987.

In general, both the first and second primary tumour were required to be malignant or intracranial to be included. However, non-melanoma malignant skin tumours were excluded from the relative risk calculations because of the known incompleteness of ascertainment in the general population and the suspected substantially better ascertainment among cases already having suffered a FPT. When an individual is suspected to have had multiple primary tumours the diagnoses of both the first and second tumours have, in over 90% of cases, been confirmed by review of the relevant pathological material. For a few cases, almost always brain tumours or retinoblastoma, where no histological material was available, confirmation of the diagnosis was based on a review of radiological or clinical evidence or both.

For tumours other than retinoblastoma treatment information was obtained from hospital and general practitioner records when readily available, but was only obtained in relation to initial treatment. Therefore treatment information for recurrence occurring a substantial time after initial treatment will have been missed.

*Statistical methods*

The method used to compare the observed number of subsequent primaries with those expected was based on the assumption that the observed number of second primaries approximated a Poisson distribution. Test of significance and confidence intervals for the relative risk of a SPT were based on exact Poisson probabilities since the numbers of events are small. Expected numbers of subsequent cancers were estimated by applying age/sex specific rates derived from cancer registration statistics (Office of Population Censuses and Surveys, 1983) to the person-years accumulated in the corresponding age/sex specific categories. The cumulative probability of a second primary tumour was estimated using standard Kaplan–Meier procedures described by Peto *et al.* (1977); comparison of the cumulative risks of SPT for different subgroups was carried out using standard tests (Peto *et al.*, 1977).

A measure of inherent risk was based on SPTs occurring among all individuals treated with neither radiotherapy nor chemotherapy; it is therefore a measure of the risk of SPT in the absence of radiotherapy or chemotherapy. It is possible that those selected for radiotherapy or chemotherapy with a particular tumour are more prone to SPTs than those not so treated. It is impossible to examine this possibility with the present data.

### Results

*Classification of tumours*

A total of 10,106 cases satisfied all criteria required for inclusion as a FPT; 90 of these developed a second primary tumour satisfying all conditions necessary for inclusion in incidence estimates. A cross-tabulation of the SPT cases is given in Table I; the rows correspond to different FPT groups classified according to morphology (World Health Organisation, 1976). The columns correspond to the different SPT groups classified firstly by site of tumour (World Health Organisation, 1969), and secondly classified by morphology. The extreme right hand column of Table I gives the total number of three-year survivors at risk of a SPT after each initial tumour type.

The comparison with expected incidence is restricted to SPTs with an ICD-8 code within the range (140-209) since this is the range where adequate general population registration figures are available. This excludes six SPTs given in Table I from the relative risk estimates, in addition to the non-melanoma malignant skin neoplasms.

*SPTs after all initial tumour types*

Of the 10,106 three-year survivors 7,871 were followed up to the end-point of the study, 31 December 1981; 2,235 were either censored (death, emigration or lost to follow-up) or were diagnosed with a SPT before the study end-point. For six of these cases no follow-up was available.

The estimates of relative risk of a second tumour after all FPTs considered together are given in Table II. A total of 78,483 person-years at risk were accumulated, and the average follow-up from three-year survival was 7.8 years. During this period 76 SPTs satisfying inclusion criteria were observed, 13 expected, yielding a relative risk about 6. The highest relative risk was for bone tumours with a relative risk of 43, and an associated 95% confidence interval from 29 to 63. In what follows similar statements are abbreviated to (RR = 43, 95%CI = 29, 63). This was followed by, in decreasing order of estimated relative risk: connective tissue (RR = 15, 95%CI = 5, 33); thyroid (RR = 14, 95%CI = 3, 41); digestive system (RR = 10, 95%CI = 5, 20) and the CNS (RR = 7, 95%CI = 4, 12). The relative risk for leukaemia was 3.2 (95%CI = 1.2, 7.0).

The estimated cumulative probability of a second primary within 25 years of three-year survival was estimated to be 3.7% (SE = 0.6%); excluding the non-melanoma skin cancer the risk was 3.3% (SE = 0.6%).

Variation in the excess risk of a SPT in relation to time elapsed from diagnosis is given in Table III. There is no clear systematic variation in relative risk with time survived, in particular there is no evidence of any substantial decrease 30 years from diagnosis. The additive excess is increasing as a consequence of an approximately constant relative risk and the general population rate of cancer increasing with follow-up.

*SPTs after retinoblastoma*

We classify unilateral cases known to be familial and bilateral cases as 'genetic', unilateral cases with no evidence of family history as 'non-genetic'. In contrast to the generality of cases included in the cohort, individuals with retinoblastoma have been followed-up much more intensively, since they have formed the basis of a separate detailed study (Draper *et al.*, 1986), and as a result we know whether radiotherapy or chemotherapy was given, at any time, for almost all cases. The majority of second primaries were osteosarcoma. Among the 366 genetic cases 23 SPTs developed; in contrast there were four SPTs among the 455 non-genetic cases.

*SPTs after genetic retinoblastoma*

*Overall risk of SPTs* The estimated cumulative risk of a SPT twenty years from three-year survival following genetic retinoblastoma was 7.9%. The percentage developing an osteosarcoma over a similar period was 6.0%. The relative risk of a malignant SPT was (RR = 29, 95%CI = 18, 43) the relative risk of a malignant bone tumour was (RR = 415, 95%CI = 232, 686); the mean follow-up period underlying these relative risks was 13.7 years. Since all the observed bone tumours were osteosarcomas, the relative risk of an osteosarcoma is much greater than 400. The relative risks of a subsequent connective tissue or central nervous system tumour were (RR = 130, 95%CI = 27, 379) and (RR = 17, 95%CI = 2, 62) respectively.

*Risk of SPT in relation to treatment* Among survivors receiving neither radiotherapy nor chemotherapy almost 3% developed a SPT within 15 years of three-year survival. The observed number of malignant SPTs was thirteen-fold expected; almost 200-fold the expected number of malignant bone tumours were observed. Details of the risks of a SPT after genetic retinoblastoma, both inherent and treatment associated, are given in Table IV. There was evidence of an

**Table I** Ninety second primary tumours classified by site and morphology

*SPT classified by ICD-8 site*

| FPT classified by ICD-0 morphology | | Salivary gland (142) | Oesophagus (150) | Large intestine except rectum (153) | Rectum (154) | Liver (155) | Peritoneum (158) | Lung (162) | Bone (170) | Connective tissue (171) | Malignant melanoma (172) | Other malig. skin (173) | Breast (174) | Cervix uteri (180) | Uterus (182) | Ovary (183) | Other urinary (189) | Brain (191) | Other CNS (192) | Thyroid gland (193) | Other endocrine glands (194) | Lymphatic leukaemia (204) | Myeloid leukaemia (205) | Other leukaemia (207) | Benign CNS (225) | Phaeochromocytoma (255) | Eosinophilic granuloma (279) |
|---|---|---|---|---|---|---|---|---|---|---|---|---|---|---|---|---|---|---|---|---|---|---|---|---|---|---|---|
| LEUKAEMIA | Lymphoid | | | | | | | | | | | | | | | | | 1 | 1 | | | | | | | | |
| | Myeloid | | | | | | | | | | | | | | | | | | 1 | | | | | | | | |
| | Other | | | | | | | | | | | | | | | | | | | | | | | | | | |
| | Unspec. | | | | | | | | | | | | | | | | | | | | | | | | | | |
| | Hodgkin's disease | | | | | | | | 2 | | | 2 | | | | | | | 1 | | | | | 1 | | | |
| | Non-Hodgkin lymphoma | | | | | | | | | | | | | | | | | | | | | | 1 | | | | |
| | Neuroblastoma and ganglioneuroblastoma | | | | | | | | | 1 | | 1 | | | | | | | | | | | | | | | |
| | Wilms' tumour | | | 2 | | | | | 1 | | | 1 | 1 | 1 | | | 1 | | | | | | 1 | | | | |
| | Retinoblastoma | | 1 | | | | 1 | | 16 | 3 | 1 | | 1 | 1 | | | | 2 | | | | | | | 1 | | |
| | Hepatoblastoma | | | | | | | | | | | | | | | | | | | | | | | | | | |
| CNS | Medulloblastoma | | | 1 | 1 | | | | 1 | | | 3 | | | | 1 | | | | 1 | | | | | 2 | | |
| | Ependymoma | | | | | | | | | | | | | | | | | | | | | | | | | | |
| | Juv. astrocytoma | 1 | | | | | | | | 1 | | 1 | | | 1 | | | | | | 1 | | 1 | | 1 | | |
| | Other astrocytoma | | | | | | | | | 2 | | | | | | | | | 1 | 1 | | | | | | | |
| | Other CNS | | | | | | | | 1 | 1 | | | | | | | | | | 1 | | | | | | | |
| | Unspec. CNS | | | | | | | | | | | | | | | | | | | | | | | | | | |
| BONE | Osteosarcoma | | | | | | | | | | | | | | | | | | | | | | | 1 | | | |
| | Ewing's tumour | | | | | | | | 1 | | | | | | | | | | | | | 1 | | | | | 1 |
| | Other malig. bone | | | | | | | | | | | | | | | | | 1 | | | | | | | | | |
| | Fibrosarcoma | | | | | | | | 1 | | | | | | | | | | | | | | | | | | |
| | Rhabdomyosarcoma | | | 1 | | | | | | | | | | | | | | 1 | | | | | | | | | |
| GERM CELL AND TROPHOBLASTIC | Malig. gonadal | | | 1 | | 1 | | | | | | | 1 | | | | | | | | | | | | | | |
| | Other malig. | | | | | | | | | | | | | | | | | | | | | | | | | | |
| | Non-malig. gonadal | | | | | | | | | | | | | | | | | | | | | | | | | | |
| | Other non-malig. | | | | | | | | | | | | | | | | | | | | | | | | | | |
| | Adrenocortical carc. | | | | | | | | | | | | | | | | | | | | | | | | | | |
| | Craniopharyngioma | | | | | | | | | | | | | | | | | | | | | | | | | | |
| | Pinealoma | | | | | | | | | | | | | | | | | | | | | | | | | | |
| | Histiocytosis X | | | | | | | | | | | | | | | | | | | | | | | | | | |
| | Other malig. neo. | | | | | | | 1 | 1 | | | | | | | | | 1 | | | | | | | | 1 | |
| | Other non-malig. neo. | | | | | | | | 2 | | | | | | | | | 2 | | | | | | | | | |
| | TOTAL SPTs | 1 | 1 | 5 | 1 | 1 | 1 | 1 | 26 | 8 | 1 | 8 | 3 | 2 | 1 | 1 | 1 | 8 | 4 | 3 | 1 | 1 | 3 | 2 | 4 | 1 | 1 |

*SPT classified by morphology*

| FPT classified by ICD-0 morphology | | Osteosarcoma | Basal cell carcinoma | Other carcinoma | Soft tissue sarcoma | Glioma | Meningioma | Acute leukaemia | Other | TOTAL SPTs | TOTAL No. 3-year survivors in each FPT category |
|---|---|---|---|---|---|---|---|---|---|---|---|
| LEUKAEMIA | Lymphoid | | | | | 2 | | | | 2 | 1850 |
| | Myeloid | | | | | | 1 | | | 1 | 163 |
| | Other | | | | | | | | | 0 | 1 |
| | Unspec. | | | | | | | | | 0 | 68 |
| | Hodgkin's disease | 2 | 2 | | | | 1 | 1 | | 6 | 840 |
| | Non-Hodgkin lymphoma | | | | | | | 1 | | 1 | 442 |
| | Neuroblastoma and ganglioneuroblastoma | | 1 | | | | | | 1 | 2 | 397 |
| | Wilms' tumour | 1 | 1 | 4 | 1 | | | 1 | | 8 | 843 |
| | Retinoblastoma | 16 | | 3 | 4 | 2 | 1 | | 1 | 27 | 821 |
| | Hepatoblastoma | | | | | | | | | 0 | 17 |
| CNS | Medulloblastoma | 1 | 3 | 3 | 1 | | 2 | | | 10 | 430 |
| | Ependymoma | | | | | | | | | 0 | 245 |
| | Juv. astrocytoma | | 1 | 2 | 1 | | 1 | 1 | 1 | 7 | 519 |
| | Other astrocytoma | | | 1 | 2 | | 1 | | | 4 | 699 |
| | Other CNS | | | 1 | 1 | | | | 1 | 3 | 316 |
| | Unspec. CNS | | | | | | | | | 0 | 134 |
| BONE | Osteosarcoma | | | | | | | 1 | | 1 | 172 |
| | Ewing's tumour | 1 | | | | | | 1 | 1 | 3 | 159 |
| | Other malig. bone | | | | | 1 | | | | 1 | 56 |
| | Fibrosarcoma | 1 | | | | | | | | 1 | 217 |
| | Rhabdomyosarcoma | | | 1 | | 1 | | | | 2 | 216 |
| GERM CELL AND TROPHOBLASTIC | Malig. gonadal | | | 3 | | | | | | 3 | 215 |
| | Other malig. | | | | | | | | | 0 | 43 |
| | Non-malig. gonadal | | | | | | | | | 0 | 28 |
| | Other non-malig. | | | | | | | | | 0 | 18 |
| | Adrenocortical carc. | | | | | | | | | 0 | 24 |
| | Craniopharyngioma | | | | | | | | | 0 | 212 |
| | Pinealoma | | | | | | | | | 0 | 25 |
| | Histiocytosis X | | | | | | | | | 0 | 75 |
| | Other malig. neo. | 1 | | 1 | | 1 | | | 1 | 4 | 681 |
| | Other non-malig. neo. | 1 | | | 1 | 2 | | | | 4 | 180 |
| | TOTAL SPTs | 24 | 8 | 19 | 11 | 9 | 7 | 6 | 6 | 90 | 10106 |

**Table II** Observed and expected SPTs among all three-year survivors

| | TOTAL | | | |
|---|---|---|---|---|
| No. in group | 10,100 | | | |
| Person-years | 78,483 | | | |
| Mean follow-up (years)[a] | 7.8 | | | |
| *SPT (ICD-8)* | *O* | *E* | *O/E* | *95%CI* |
| All sites (140–209)[b] | 76 | 13.06 | 5.8 | (4.6, 7.2) |
| Digestive (150–159) | 9 | 0.87 | 10.3 | (4.7, 19.6) |
| Bone (170) | 28 | 0.65 | 43.3 | (28.9, 62.6) |
| Connective tissue (171) | 6 | 0.40 | 15.1 | (5.5, 32.8) |
| Breast (174) | 3 | 1.06 | 2.8 | (0.6, 8.2) |
| Genito-urinary (180–189) | 5 | 2.40 | 2.1 | (0.7, 4.9) |
| CNS (191–192) | 12 | 1.72 | 7.0 | (3.6, 12.2) |
| Thyroid (193) | 3 | 0.21 | 14.1 | (2.9, 41.2) |
| Leukaemia (204–207) | 6 | 1.87 | 3.2 | (1.2, 7.0) |

[a]In this table and in all subsequent tables where mean follow-up intervals are quoted they exclude the initial 3 years all subjects need to survive to be included in the present study; [b]In this table and all subsequent tables providing relative risks for 'all sites', non-melanoma malignant skin tumours are excluded from both observed and expected numbers.

increased risk of SPTs generally and osteosarcoma in particular following radiotherapy without chemotherapy compared to that following neither radiotherapy nor chemotherapy. Furthermore there is suggestive evidence that the risks of SPTs generally, and osteosarcoma in particular, are increased following radiotherapy and chemotherapy (almost always cyclophosphamide in this series) compared to those given radiotherapy but no chemotherapy. However, cases given chemotherapy were also more likely to have had more than one course of radiotherapy and radioactive implants. Thus although there is some evidence of an increased risk of SPT associated with the use of cyclophosphamide, this may be due to cyclophosphamide, radiotherapy or an interaction between them. For a more detailed examination of the associations between risk of SPT and therapy the reader should consult our more detailed papers on retinoblastoma (Draper *et al.*, 1986; Hawkins, 1987).

### *SPTs after all FPTs except retinoblastoma*

For the sub-cohort of all FPTs except retinoblastoma we give, in Table V, the numbers of three-year survivors at risk following treatment with radiotherapy, chemotherapy, both and neither of these forms of therapy. Two important considerations are illustrated by Table V.

**Table V** Treatment given for FPTs other than retinoblastoma

| | *No. at risk* | *No. SPTs* | *No. SPTs Inside/edge RT field* | *No. of SPT leuks.* |
|---|---|---|---|---|
| RT or CH no record | 1,656 | 1 | 0 | 0 |
| Neither RT nor CH | 1,495 | 10 | — | 0 |
| RT but no CH | 2,668 | 40 | 26 | 3 |
| CH but no RT | 767 | 1 | — | 0 |
| Both RT and CH | 2,699 | 11 | 6 | 3 |
| | 9,285 | 63 | 32 | 6 |

Firstly there is a relatively small number of SPTs after chemotherapy and thus we are unable to examine the risk of SPT associated with chemotherapy in much detail following specific FPTs. This arises from the relatively recent widespread use of chemotherapy, and, given the current study end-point, average follow-up times are short. The mean follow-up times beyond three years for those having received neither radiotherapy nor chemotherapy, radiotherapy but no chemotherapy and both radiotherapy and chemotherapy following FPTs other than retinoblastoma were 9.5, 9.8 and 3.8 years respectively (see Table VII).

The second general point illustrated by Table V follows from the large category of cases with no record of whether radiotherapy or chemotherapy were given. Comparisons between different treatment subgroups must be undertaken carefully since the records of a patient who develops a SPT are pursued with much greater effort than are those for cohort members not developing a SPT; as a result we know the details with regard to both radiotherapy and chemotherapy for all but one of the patients developing a SPT.

**Table III** Excess risk of SPT at varying times from diagnosis

| | *Completed years from diagnosis* | | | | | | |
|---|---|---|---|---|---|---|---|
| | *3–4* | *5–9* | *10–14* | *15–19* | *20–24* | *25–29* | *30–* |
| Person-years (PY) | 17,961 | 27,995 | 16,237 | 9,350 | 4,580 | 1,650 | 710 |
| Observed SPTs (O) | 9 | 25 | 17 | 8 | 6 | 6 | 5 |
| Expected SPTs (E) | 1.8 | 3.1 | 2.5 | 2.2 | 1.6 | 0.9 | 0.9 |
| Relative risk (O/E) | 4.9 | 8.0 | 6.8 | 3.6 | 3.7 | 6.8 | 5.7 |
| Additive excess risk [(O–E)/PY] 1000 | 0.4 | 0.8 | 0.9 | 0.6 | 1.0 | 3.1 | 5.8 |

**Table IV** Risk of SPT after genetic retinoblastoma – Association with treatment

| *Treatment group* | *No.* | *Mean follow-up period (yrs)* | *SPT Type* | *SPT No.*[a] | *% with SPT by 15 yrs from 3-year survival (standard error)* | *O*[b] | *E* | *O/E* | *95%CI* |
|---|---|---|---|---|---|---|---|---|---|
| Neither | 60 | 13.4 | all | 2 | 2.7% (3.3%) | 2 | 0.15 | 13 | (2, 47) |
| RT nor CH | | | bone | 1 | 2.7% (3.3%) | 1 | 0.01 | 174 | (4, 965) |
| RT but no CH | 241 | 13.8 | all | 13 | 6.8% (2.5%) | 13 | 0.49 | 26 | (14, 45) |
| | | | bone | 8 | 5.2% (2.2%) | 8 | 0.02 | 340 | (146, 668) |
| Both RT | 62 | 13.5 | all | 7 | 13.3% (5.8%) | 7 | 0.09 | 78 | (31, 160) |
| and CH | | | bone | 5 | 9.2% (5.0%) | 5 | 0.01 | 771 | (250, 1802) |

[a]Total number of SPTs observed; [b]Total number of observed SPTs eligible for inclusion in the relative risk.

Hence, if we analyse any group of patients for which the treatment is known we obtain spuriously high estimates of incidence, the amount of possible bias being determined by the size of the 'no record' category. All subsequent analyses were carried out separately, including and excluding the 'no-record' category. However, we report the results of both only when inclusion or exclusion affects the risk estimates materially; otherwise we report the results excluding the 'no-record' category.

Some of the individuals developing a SPT were known to have a genetic condition known to predispose to neoplasia, the most common condition being neurofibromatosis; there were also some individuals with Gorlin's (basal cell naevus) syndrome, and one from a family with Multiple Endocrine Neoplasia type 2 (MEN2 or Sipple's syndrome). In all results that follow we have carried out the analysis both including and excluding these cases. We again report the results of both analyses when inclusion or exclusion materially affected the risk estimates, otherwise we report the risk including these individuals.

*Risk of SPT following all FPTs except retinoblastoma*

*Overall risk of SPT* We observed almost five-fold the number of malignant tumours expected, Table VI. The number of malignant bone tumours observed was almost twenty-fold expected, eight osteosarcomas being observed among the ten malignant bone tumours. All thyroid and all but one of the connective tissue tumours arose after a CNS tumour and are discussed below. Almost ten-fold the expected number of digestive tract tumours were observed. Seven-fold the expected number of CNS tumours were observed and four-fold the expected number of leukaemias.

The cumulative risk of a SPT by 25 years from three-year survival was 3.7% (SE = 0.8%).

**Table VI** Observed and expected SPTs among three-year survivors of all FPTs except retinoblastoma

| | TOTAL |
|---|---|
| No. in group | 9,279 |
| Person-years | 67,449 |
| Mean follow-up (years) | 7.3 |

| *SPT (ICD-8)* | *O* | *E* | *O/E* | *95%CI* |
|---|---|---|---|---|
| All sites (140–209) | 50 | 11.20 | 4.5 | (3.3, 5.9) |
| Digestive (150–159) | 7 | 0.74 | 9.5 | (3.8, 19.6) |
| Bone (170) | 10 | 0.57 | 17.7 | (8.5, 32.5) |
| Connective tissue (171) | 5 | 0.35 | 14.4 | (4.7, 33.7) |
| Breast (174) | 2 | 0.86 | 2.3 | (0.3, 8.4) |
| Genito-urinary (180–189) | 4 | 2.08 | 1.9 | (0.5, 4.8) |
| CNS (191–192) | 10 | 1.57 | 6.8 | (3.3, 12.5) |
| Thyroid (193) | 3 | 0.19 | 16.0 | (3.3, 46.9) |
| Leukaemia (204–207) | 6 | 1.59 | 3.8 | (1.4, 8.2) |

*Inherent risk of SPT* By twenty years from three-year survival among those given neither radiotherapy nor chemotherapy the risk of a SPT is under 2%, Table VII. This corresponds to about four-fold the number of subsequent malignant tumours expected. Among these individuals the risk of a subsequent bone or CNS tumour is greater than expected, although because of small numbers the magnitude of the excess is subject to wide confidence limits in both cases.

*Risk of SPT in relation to radiotherapy* Among those given radiotherapy but no chemotherapy the cumulative risk of a SPT within twenty years of three-year survival was just under 3%, see Table VII. A comparison of the cumulative risk of a SPT following radiotherapy but no chemotherapy with the inherent cumulative risk revealed they were significantly different at the $P = 0.02$ level (one-sided test) irrespective of inclusion or exclusion of the genetically predisposed cases developing a SPT. Twenty-nine of the forty SPTs arose inside or on the edge of tissue directly irradiated to treat the FPT, this includes three second leukaemias. Of the eleven SPTs arising outside tissue directly irradiated four arose in individuals with neurofibromatosis.

Although specifically excluded from the relative risks there were eight non-melanoma malignant skin tumours, all basal cell carcinomas. All developed within tissue directly irradiated to treat the FPT. Seven of the eight were diagnosed more than a decade after radiotherapy for the FPT. Among those given radiotherapy but no chemotherapy the cumulative risk of a basal cell carcinoma was 0.7% (SE = 0.4%) by twenty years from three-year survival. One of the eight, a patient with Gorlin's (basal cell naevus) syndrome, was genetically predisposed.

In all we observed six-fold the number of malignant SPTs expected and about twenty-fold the number of malignant bone tumours expected; four were osteosarcomas arising within tissue directly irradiated. There were seven malignant digestive tract tumours observed among all three-year survivors excluding retinoblastoma, six of these occurring among those given radiotherapy but not chemotherapy; within this treatment subgroup this represents fifteen-fold the number expected. None of these individuals was known to have genetic conditions predisposing to neoplasia. Furthermore three of these digestive tract SPTs arose within directly irradiated tissue. Three second primary leukaemias developed following radiotherapy but no chemotherapy; this represents five-fold the number expected.

There is no clear systematic variation in relative risk with increasing follow-up.

*Risk of SPT in relation to radiotherapy and chemotherapy* The mean follow-up period following both radiotherapy and chemotherapy was substantially less than for those with treatments not involving chemotherapy, Table VII; therefore our results necessarily apply to this shorter interval. The cumulative risk of a SPT by ten years from three-year

**Table VII** Risk of SPT after all FPTs except retinoblastoma – Association with treatment

| *Treatment group* | *No.* | *Mean follow-up period (yrs)* | *SPT No.*[a] | *% with SPT by specified time from 3-year survival (standard error)* | | $O^b$ | *E* | *O/E* | *95%CI* |
|---|---|---|---|---|---|---|---|---|---|
| Neither RT nor CH | 1495 | 9.5 | 10 | 1.6% (0.9%) | by 20 yrs | 10 | 2.6 | 3.9 | (1.9, 7.1) |
| RT but no CH | 2668 | 9.8 | 40 | 2.7% (0.8%) | by 20 yrs | 29 | 5.1 | 5.6 | (3.8, 8.1) |
| Both RT and CH | 2699 | 3.8 | 11 | 0.6% (0.7%) | by 10 yrs | 10 | 1.1 | 9.3 | (4.5, 17.1) |

[a]Total number of SPTs observed; [b]Total number of observed SPTs eligible for inclusion in the relative risk.

survival was under 1%, Table VII. The observed number of subsequent malignant tumours was about ten-fold expected. However, this was reduced to four-fold expected with inclusion of all the possible 'no record' cases. The observed number of subsequent primary leukaemias was five to ten-fold expected depending on whether the 'no record' cases were included or excluded.

*SPTs after CNS tumours*

The cumulative risk of a SPT by twenty years from three-year survival is 2.4% (SE = 1.1%). However, because of the occurrence of many SPTs after twenty years this rises to 5.1% (SE = 3.7%) by 25 years from three-year survival. Relative risks of subsequent malignant tumour are reported in Table VIII; seventeen SPTs were observed, 3.37 expected, yielding a relative risk of about five. There was a larger than expected number of both malignant connective tissue and thyroid tumours.

**Table VIII** Observed and expected SPTs among three-year survivors of CNS tumours

| | TOTAL | | | |
|---|---|---|---|---|
| No. in group | 2,341 | | | |
| Person-years | 19,325 | | | |
| Mean follow-up (years) | 8.3 | | | |
| *SPT (ICD-8)* | *O* | *E* | *O/E* | *95%CI* |
| All sites (140–209) | 17 | 3.37 | 5.0 | (2.9, 8.1) |
| Digestive (150–159) | 2 | 0.23 | 8.5 | (1.0, 30.8) |
| Bone (170) | 2 | 0.17 | 12.0 | (1.4, 43.3) |
| Connective tissue (171) | 4 | 0.10 | 38.8 | (10.6, 99.3) |
| Breast (174) | 0 | 0.29 | | |
| Genito-urinary (180–189) | 2 | 0.66 | 3.1 | (0.4, 11.0) |
| CNS (191–192) | 1 | 0.41 | 2.4 | (0.1, 13.5) |
| Thyroid (193) | 3 | 0.06 | 48.7 | (10.0, 142.2) |
| Leukaemia (204–207) | 1 | 0.43 | 2.3 | (0.1, 12.9) |

*Inherent risk of SPT* Among survivors receiving neither radiotherapy nor chemotherapy the incidence of subsequent malignant tumours was very similar to that expected, see Table IX. In fact among these survivors just one SPT was observed; the individual concerned had neurofibromatosis.

*Risk of SPT in relation to radiotherapy* The cumulative risk of a SPT by twenty years from three-year survival among those given radiotherapy but no chemotherapy was 4.4%; if individuals known to be genetically predisposed are excluded the corresponding figure is 3.2%; see Table IX. Thirteen of the twenty-one SPTs arose within irradiated tissue, this includes one leukaemia. Of the eight SPTs developing outside irradiated tissue, four arose in individuals with neurofibromatosis. We observed almost nine-fold the number of subsequent malignant tumours expected. The excess was reduced to just over six-fold when individuals genetically predisposed were excluded. A comparison of the cumulative risk of SPT after neither radiotherapy nor chemotherapy with that after radiotherapy but no chemotherapy was significant ($P = 0.0002$ including the genetically predisposed; $P = 0.0003$ excluding the genetically predisposed). This provides evidence of radiotherapy being involved in the development of the excess of SPT observed following CNS tumours. However, very few three-year survivors of medulloblastoma or ependymoma had not received radiotherapy, and therefore the measure of inherent risk is based almost entirely on survivors of astrocytoma and CNS tumours other than medulloblastoma and ependymoma.

The four malignant connective tissue tumours observed following radiotherapy but not chemotherapy all arose in individuals with neurofibromatosis; also each SPT arose well outside the volume of tissue directly irradiated to treat the original CNS tumour. Each of the thyroid carcinomas arose inside the tissue directly irradiated to treat the original CNS tumour. Among those treated with radiotherapy but no chemotherapy the relative risk of a malignant thyroid tumour was (RR = 108, 95%CI = 22, 316).

In summary, there is no evidence of the inherent risk of a SPT following a CNS tumour being much different from that expected. However, there is evidence of an increased absolute and relative risk attributable to treatment by radiotherapy without chemotherapy, given that a majority of SPTs developed within tissue directly irradiated, and taking into account the histological types of SPT, and the intervals between radiotherapy and diagnosis of the SPTs.

The relative risk of a SPT among these patients is about ten after a decade from diagnosis and again there is no evidence of decline in the relative risk with increased survival time.

*SPTs after Wilms' tumour*

The cumulative risk of a SPT by 20 years from three-year survival is 3.1% (SE = 1.9%). The relative risk of a subsequent malignant tumour was (RR = 8, 95%CI = 3, 16).

**Table IX** Risk of SPT after central nervous system tumours – Association with treatment

| *Treatment* | | *No.* | *Mean follow-up period (yrs)* | *SPT Site* | *SPT No.*[a] | *% with SPT by 20 years from 3-yr survival (standard error)* | *O*[b] | *E* | *O/E* | *95%CI* |
|---|---|---|---|---|---|---|---|---|---|---|
| Neither RT nor CH | Including genetically predisposed SPT cases | 578 | 9.0 | all | 1 | 0.7% (0.9%) | 1 | 1.0 | 1.0 | (0.0, 5.6) |
| | Excluding genetically predisposed SPT cases | 577 | 9.0 | all | 0 | 0.0% | 0 | | | |
| RT but no CH | Including genetically predisposed SPT cases | 1106 | 8.0 | all | 21 | 4.4% (1.9%) | 14 | 1.6 | 8.6 | (4.7, 14.3) |
| | Excluding genetically predisposed SPT cases | 1101 | 8.0 | all | 16 | 3.2% (1.6%) | 10 | 1.6 | 6.2 | (3.0, 11.3) |

[a]Total number of SPTs observed; [b]Total number of observed SPTs eligible for inclusion in the relative risk.

*Risk of SPT in relation to radiotherapy* Among those given radiotherapy but no chemotherapy there was a cumulative risk of SPT of just under 4% by twenty years from three-year survival. Six of the seven SPTs developed in tissue directly irradiated. The relative risk of a subsequent malignant tumour was (RR = 12, 95%CI = 4, 26) based on an average follow-up period of over 18 years from three-year survival.

Considering the histological types of SPT, the period elapsed after radiotherapy before SPTs appear is at least 13 years, and all SPTs except one arise within tissue irradiated, we conclude it is likely that radiation is involved in the development of the excess of SPTs observed.

*SPTs after Hodgkin's disease*

The cumulative risk of a SPT by ten years from three-year survival is estimated to be 1.3% (SE = 0.6%). The relative risk of a subsequent malignant tumour was approximately four; this was significantly larger than one (one-tailed test, $P = 0.027$), and there was an excess of bone tumours – both osteosarcomas.

*Risk of SPT in relation to radiotherapy* Among those receiving radiotherapy but no chemotherapy the cumulative risk of a SPT by ten years from three-year survival was 1.5% (SE = 0.9%). All four SPTs developed within tissue directly irradiated. The relative risk of a malignant bone tumour was (RR = 64, 95% CI = 8,231) and again the relative risk of osteosarcoma would be greater.

*SPTs after leukaemia*

Only three SPTs, all CNS, were observed, though the mean follow-up period beyond three-years was only 3.4 years. As a consequence our results following leukaemia are very provisional. Nevertheless the number of subsequent malignant CNS tumours observed was about twenty-fold expected.

### Discussion

It is not the purpose of the present study to examine the relationship between risk of SPT and treatment in detail; this will be undertaken in a case-control study in which exhaustive efforts will be made to obtain all treatment information. However, we examine this relationship in rather broad terms.

It is very difficult to compare the overall risk from different series because of the many confounding factors, including: different series compositions with regard to the initial childhood cancers, different treatments, different entry to risk points, different follow-up methods and assumptions and different average follow-up periods. However, we shall attempt to compare the overall risks in general terms. In our series the estimated cumulative probability of SPT by 25 years from three-year survival was about 4%, the relative risk of a SPT was about six corresponding to an average follow-up period of about eight years from three-year survival. The relative and cumulative risks of a SPT were estimated to be substantially higher in the reports from the Sidney Farber Cancer Institute (SFCI) series (Li *et al.*, 1975; Li, 1977): a relative risk of over twenty among five-year survivors was reported, the cumulative probability of a second malignancy was estimated to be 12% (SE = 4%) by twenty years from five-year survival; among those who received orthovoltage radiotherapy the cumulative risk of a second primary cancer within the radiation field was 17% (SE = 6%) twenty years from five-year survival. The latest incidence report from the Late Effects Study Group (LESG) (Tucker *et al.*, 1984) gives a relative risk of fifteen corresponding to an average follow-up of 5.5 years; the cumulative risk was estimated to be 12% by 25 years from diagnosis. Even in the initial report on incidence from LESG (Mike *et al.*, 1982) where retinoblastoma was specifically excluded, the risks are no smaller than in our study. The risks from the University of Minnesota series of children treated with megavoltage radiotherapy (Potish *et al.*, 1985) were higher than in our series but they include many benign tumours that we would exclude from consideration.

The relative risk of SPT remained fairly constant with time survived in our series, six-fold expected, for the first 30 years following three-year survival after childhood cancer. There is no evidence of the risk diminishing with increasing time from diagnosis; this is in agreement with Tucker *et al.* (1984).

Radiotherapy was considered to have contributed substantially towards the excess of second tumours observed in the LESG, SFCI and the University of Minnesota series. The increased risk of SPTs in relation to radiotherapy compared to inherent risk in our study provides evidence that radiotherapy is involved in the development of some of the excess of SPTs observed following childhood cancer; particularly following retinoblastoma and central nervous system tumours. This interpretation is supported by the following: the histological types of SPTs observed; the proportion of SPTs developing within tissue directly irradiated to treat the FPT; the intervals between radiotherapy and diagnosis of the SPTs.

Although there is substantial evidence from these studies of a relationship between radiotherapy and an increased risk of SPTs there is comparatively little evidence available on the risk associated with chemotherapy given to treat cancer in children. At present, within our cohort the average follow-up times beyond three years are short following chemotherapy except among retinoblastoma survivors. However, some indication of what may emerge in future analyses may be obtained from the accompanying paper (Kingston *et al.*, 1987), since this contains cases excluded from the cohort analysis because either the FPT or SPT occurred too recently for inclusion. We note here only that there is evidence that types of FPT and SPT observed appear to be changing, in that among individuals with their FPT diagnosed since 1970 the number of survivors subsequently developing leukaemia has increased substantially, the increase being spread across several FPT types including central nervous system tumours, acute leukaemia and lymphoma. This period corresponds to the increasing use of intensive multiple agent chemotherapy and it is tempting to consider chemotherapy as a cause. This might be indirect for instance as a result of immunosuppression, or because chemotherapy provided sufficient survival time for SPTs to emerge as a result of a mechanism not directly involving the chemotherapy, for example inherent predisposition (Penn, 1982).

A recent paper by the LESG (Tucker *et al.*, 1985*a*) reports an excess of second primary leukaemia within a cohort of individuals surviving at least two years from diagnosis of childhood cancer (O = 22, E = 1.52, O/E = 14, 95%CI = (9, 22)). To determine if the increased risk was related to therapy a case-control study was carried out. No increase in risk was associated with radiotherapy. However, there was a significant relationship between dose of alkylating agents and the relative risk of secondary leukaemia, the relative risk reaching about twenty for the high (alkylating agent) dose categories. They conclude that the excess risk of secondary leukaemia following childhood cancer was almost entirely due to alkylating agents.

Genetic predisposition to multiple primary tumours exists among the survivors of genetic retinoblastoma, and the individuals with neurofibromatosis or Gorlin's (basal cell naevus) syndrome who developed a FPT of the central nervous system followed by a variety of SPTs. Among the survivors of genetic retinoblastoma there was an inherent risk of SPTs about thirteen-fold that expected from general population rates of cancer; the inherent risk of osteosarcoma exceeding 174-fold expected. After all first primary tumours except retinoblastoma, considered as one group, there was an inherent risk of a subsequent malignant tumour about four-fold expected.

*Second primary tumours following retinoblastoma*

In our study the overall relative risk of a SPT was about 30 following genetic retinoblastoma, observed malignant bone tumours being more than 400-fold expected. There was evidence of an increased risk of SPT generally and osteosarcomas in particular following radiotherapy for genetic retinoblastoma compared to the inherent risk. There is some evidence of an increased risk of SPT associated with the use of cyclophosphamide, though it is not possible to be sure whether this is due to cyclophosphamide alone or radiotherapy or an interaction between them.

From a series of individuals on file at the Ophthalmic Oncology Center of the New York Hospital, Cornell Medical Center, a report (Abramson *et al.*, 1984) gives risks of 20%, 50% and 90% at 10, 20 and 30 years from diagnosis of genetic retinoblastoma respectively; also reported are risks of second tumours in patients treated without radiotherapy or where tumours developed outside the radiation field of 10%, 30% and 68% at 10, 20 and 32 years from diagnosis respectively. These cumulative risks are an order of magnitude greater than estimated from our series, and it is very hard to explain the large discrepancy. However, we are satisfied that we have not missed a substantial number of SPTs within our cohort. Another large series is part of the LESG report (Tucker *et al.*, 1984), where 319 two-year survivors of retinoblastoma have been followed-up for an average period of seven years. No separation of unilateral and bilateral cases was carried out; the overall relative risk of a second primary was about 60; bone tumours were 1,000-fold expected.

*Second primary bone tumours following childhood cancer other than retinoblastoma*

After all first primary tumours except retinoblastoma, considered as one group, the number of malignant bone tumours observed was eighteen-fold expected. Eight were osteosarcomas, one a fibrosarcoma and one an unspecified malignant tumour. Six of the bone tumours arose within directly irradiated tissue. The LESG has reported results from a case-control study of the involvement of radiotherapy and chemotherapy in the development of second primary bone cancer following childhood cancer (Tucker *et al.*, 1985*b*). In both retinoblastoma and other patients, the relative risk increased with increasing radiation dose, reaching a value of almost 40 at doses over 50 Gy. Independently of radiotherapy, alkylating agents were associated with a significant two-fold risk of bone cancer in both retinoblastoma and other patients.

*Second primary CNS tumours following childhood cancer other than retinoblastoma*

The number of second primary malignant CNS tumours observed was about seven-fold expected. The presence of an increased inherent risk among survivors of childhood cancer is in line with the report of an increased likelihood of CNS tumours, leukaemia, and childhood tumours in relatives of children with CNS neoplasms (Farwell & Flannery, 1984*b*). Several familial cancer syndromes have been reported involving tumours of the CNS; two, Turcot's syndrome (Turcot *et al.*, 1959; Todd *et al.*, 1981) and the Li–Fraumeni syndrome (Li & Fraumeni, 1969; Li & Fraumeni, 1982; Birch *et al.*, 1984), are well documented. Meadows *et al.* (1977) have suggested that the frequency of CNS tumours with leukaemia in their series may indicate a hereditary cancer syndrome.

*Basal cell carcinoma following childhood cancer other than retinoblastoma*

Eight basal cell carcinomas were observed following a variety of FPTs, all within tissue directly irradiated to treat the FPT; one individual was known to have Gorlin's (basal cell naevus) syndrome; all but one of the cases occurred more than a decade from radiotherapy, all evidence consistent with the involvement of radiation. Previous reports have implicated radiotherapy in the development of skin carcinomas following childhood cancer, notably Meadows *et al.* (1985).

*SPTs following CNS tumours*

Given the evidence of a familial element in cancer occurring in some families with a child with CNS tumour, it would not have been surprising to have identified an increased inherent risk of SPT above that expected. However, there was no evidence from our series of a substantially increased inherent risk of SPT among survivors of a CNS tumour. Of course we cannot exclude the possibility of there being a small excess inherent risk not detectable with current numbers of tumours. From our cohort there is convincing prima facie evidence that radiotherapy is involved in the development of an excess of subsequent malignant tumours following a CNS tumour, six to nine-fold expected depending on whether genetically predisposed cases are included or excluded. As might have been anticipated from previous work (Shore *et al.*, 1984; Ron & Modan, 1984) there was an excess of thyroid cancer, 100-fold expected among those treated with radiotherapy but no chemotherapy.

In a series of 670 individuals diagnosed with a CNS neoplasm before age twenty, nine had another primary neoplasm either before or after the CNS primary (Farwell & Flannery. 1984*a*). The expected number of neoplasms was 0.99, yielding a relative risk of about nine. Radiotherapy was identified as a possible contributing factor to the development of the SPT in four individuals.

*SPTs after Wilms' tumour*

The LESG (Tucker *et al.*, 1984) examined the observed and expected numbers of second primary cancers among 1,248 children surviving at least two years from diagnosis of Wilms' tumour: the relative risk of any subsequent cancer was 24. Relative risks were significantly raised for thyroid, bone, connective tissue, digestive tract, CNS and leukaemia.

Another large study of survivors of Wilms' tumour (Li *et al.*, 1983), was based on 487 individuals treated at the Dana–Farber Cancer Institute and Children's Hospital of Boston. Thirty SPTs were observed: eleven malignant, sixteen benign and three border line neoplasms. The cumulative risk of a second tumour was 6% (SE = 2%) by twenty years from diagnosis. All but one second malignant tumour arose within the prior radiotherapy field. Of the benign second tumours nine were within the radiotherapy field. Among those given radiotherapy the relative risk of a second cancer was fourteen.

The results from the Dana–Farber series are similar to those from our series, though the risks from the LESG series are much larger.

*SPTs after leukaemia*

No firm conclusions may yet be drawn concerning the SPT risk following leukaemia in our series, since the average follow-up period beyond three-year survival is only just over three years. The twenty-fold excess of CNS tumours is quite striking, though it is based on only three cases. These all arose in individuals treated with chemotherapy and CNS irradiation; we cannot infer that treatment was necessarily directly involved in their development, since we have no measure of the risk of SPT in the absence of therapy.

*Conclusion*

The rate of occurrence of SPT is low and the risk attributable to therapy is small when compared to the improvements in survival rates achieved by the use of these treatments. Our data confirm that the use of radiotherapy is associated with an increased risk of SPT. However, the risks of SPT in our data are almost always lower than those reported from series treated in the United States. It is possible that the lower frequency of SPT observed in our data may be accounted for, to some extent, by the use of less combination therapy and lower doses of radiotherapy in Britain. It is our intention to explore these international differences further in the future. It is important to continue to monitor survivors so that tumour and treatment combinations giving rise to particularly excessive frequencies of SPTs may be identified and alternative therapies examined to see whether comparable survivla may be achieved with reduced adverse late effects.

Initially sincere thanks to Dr M. Kinnier Wilson for her help and suggestions in the preparation of this paper.

We thank Prof H.B. Marsden for reviewing the histology. Particular thanks to Elizabeth Mowat, Michael Potok, Barbara Sanders, Rupert Smith and David Winter for their useful contributions.

We are grateful to Mr C.W.P. Fearnley and to Mrs E.M. Roberts for their work on the National Registry of Childhood Tumours.

We thank the members of the Long Term Follow-up Study Working Party (Prof D. Harnden, Dr J. Mann, Prof J. Malpas, Prof H.B. Marsden, Dr P. Morris Jones and Dr D. Pearson) for their advice and support on this study and them, and many other consultants, for access to medical research records concerning their patients.

The Long Term Follow-up Study of childhood cancer survivors is supported by the Cancer Research Campaign and the Leukaemia Research Fund. The Childhood Cancer Research Group is supported by the Department of Health and Social Security and the Scottish Home and Health Department.

We are grateful to the Office of Population Censuses and Surveys, the Information Services Division of the Common Services Agency of the Scottish Health Service, the Registrar General for Scotland, and regional cancer registries for providing copies of notifications of childhood cancer cases. We thank the National Health Service Registers at Southport and Edinburgh for notification of deaths, cancers and the 'flagging' of survivors.

**References**

ABRAMSON, D.H., ELLSWORTH, R.M., KITCHIN, F.D. & TUNG, G. (1984). Second nonocular tumours in retinoblastoma survivors: are they radiation-induced? *Ophthalmology*, **91**, 1351.

BIRCH, J.M., HARTLEY, A.L., MARSDEN, H.B., HARRIS, M. & SWINDELL, R. (1984). Excess risk of breast cancer in the mothers of children with soft tissue sarcomas. *Br. J. Cancer*, **49**, 325.

BOICE, J.D. (1981). Cancer following medical irradiation. *Cancer*, **47**, 1081.

COMMITTEE ON THE BIOLOGICAL EFFECTS OF IONIZING RADIATIONS (BEIR). (1980). *The effects on populations of exposure to low levels of ionizing radiation*. National Academy Press, Washington, D.C., USA.

DRAPER, G.J., BIRCH, J.M., BITHELL, J.F. & 6 others (1982). *Childhood cancer in Britain; Incidence, survival and mortality*. Office of Population Censuses and Surveys, Studies on Medical and Population Subjects, No. 37.

DRAPER, G.J., SANDERS, B.M. & KINGSTON, J.E. (1986). Second primary neoplasms in patients with retinoblastoma. *Br. J. Cancer*, **53**, 661.

FARWELL, J. & FLANNERY, J.T. (1984*a*). Second primaries in children with central nervous system tumours. *J. Neuro. Oncol.*, **2**, 371.

FARWELL, J. & FLANNERY, J.T. (1984*b*). Cancer in relatives of children with central nervous system neoplasms. *New Engl. J. Med.*, **311**, 749.

HAWKINS, M.M. (1987). Second primary tumours among childhood cancer survivors treated with anti-cancer drugs. In *Carcinogenicity of Alkylating Cytostatic Drugs*, Schmahl, D. & Kaldor, J.M. (eds) p. 231. IARC Scientific Publications, 78.

INTERNATIONAL AGENCY FOR RESEARCH ON CANCER (1981). IARC monographs on the evaluation of the carcinogenic risk of chemicals to humans: Some antineoplastic and immunosuppressive agents. Vol. **26**.

KINGSTON, J.E., HAWKINS, M.M., DRAPER, G.J., MARSDEN, H.B. & KINNIER WILSON, L.M. (1987). Patterns of multiple primary tumours in patients treated for cancer during childhood. *Br. J. Cancer*, **56**, 331.

LI, F.P. & FRAUMENI, J.F. (1969). Soft tissue sarcomas, breast cancer, and other neoplasms: A familial syndrome? *Ann. Intern. Med.*, **71**, 747.

LI, F.P., CASSADY, J.R. & JAFFE, N. (1975). Risk of second tumours in survivors of childhood cancer. *Cancer*, **35**, 1230.

LI, F.P. (1977). Second malignant tumours after cancer in childhood. *Cancer*, **40**, 1899.

LI, F.P. & FRAUMENI, J.F. (1982). Prospective study of a family cancer syndrome. *JAMA*, **247**, 2692.

LI, F.P., CAI-JIE YAN, J., SALLAN, S. & 5 others (1983). Second neoplasms after Wilms' tumour in childhood. *J. Natl Cancer Inst.*, **71**, 1205.

MEADOWS, A.T., BAUM, E., FOSSATI-BELLANI, F. & 10 others (1985). Second malignant neoplasms in children: An update of the Late Effects Study Group. *J. Clin. Oncol.*, **3**, 532.

MEADOWS, A.T., D'ANGIO, G.J., MIKE, V. & 4 others (1977). Patterns of second malignant neoplasms in children. *Cancer*, **40**, 1903.

MIKE, V., MEADOWS, A.T. & D'ANGIO, G.J. (1982). Incidence of second malignant neoplasms in children: Results of an international study. *Lancet*, **ii**, 1326.

OFFICE OF POPULATION CENSUSES AND SURVEYS (1983). *Cancer registration surveillance 1968–1978 England and Wales*. OPCS, London.

PENN, I. (1982). Mechanisms of therapy-induced malignancies – editorial review. *Cancer Surveys*, **1**, 763.

PETO, R., PIKE, M.C., ARMITAGE, P. & 7 others (1977). Design and analysis of randomized clinical trials requiring prolonged observation of each patient. *Br. J. Cancer*, **35**, 1.

POTISH, R.A., DEHNER, L.P., HASELOW, R.E., KIM, T.H., LEVITT, S.H. & NESBIT, M. (1985). The incidence of second neoplasms following megavoltage radiation for paediatric tumours. *Cancer*, **56**, 1534.

RON, E. & MODAN, B. (1984). Thyroid and other neoplasms following childhood scalp irradiation. In *Radiation Carcinogenesis: Epidemiology and Biological Significance*, Boice, J.D. & Fraumeni, J.F. (eds) p. 139. Raven Press: New York.

SCHMAHL, D., HABS, M., LORENZ, M. & WAGNER, I. (1982). Occurrence of second tumours in man after anti-cancer drug treatment. *Cancer Treatment Reviews*, **9**, 167.

SHORE, R.E., WOODWARD, E. & HEMPELMANN, L.H. (1984). Radiation-induced thyroid cancer. In *Radiation Carcinogenesis: Epidemiology and Biological Significance*, Boice, J.D. & Fraumeni, J.F. (eds) p. 131. Raven Press: New York.

STILLER, C.A. (1985). Descriptive epidemiology of childhood leukaemia and lymphoma in Great Britain. *Leukaemia Research*, **9**, 671.

TODD, D.W., CHRISTOFERSON, L.A., LEECH, R.W. & RUDOLF, L. (1981). A family affected with intestinal polyposis and gliomas. *Ann. Neurol.*, **10**, 390.

TUCKER, M.A., MEADOWS, A.T., BOICE, J.D., HOOVER, R.N. & FRAUMENI, J.F. (1984). Cancer risk following treatment of childhood cancer. In *Radiation Carcinogenesis: Epidemiology and Biological Significance*, Boice, J.D. & Fraumeni, J.F. (eds) p. 211. Raven Press: New York.

TUCKER, M.A., MEADOWS, A.T., BOICE, J.D. & 7 others (1985*a*). Leukaemia after therapy with alkylating agents for childhood cancer. *J. Natl Cancer Inst.*, **78**, 459.

TUCKER, M.A., MEADOWS, A.T., BOICE, J.D. & 4 others (1985*b*). Bone cancer linked to radiotherapy and chemotherapy in children. *Proc. Amer. Soc. Clin. Oncol.* (Abstract).

TURCOT, J., DESPRES, J.P. & ST. PIERRE, F. (1959). Malignant tumours of the central nervous system associated with familial polyposis of the colon: Report of two cases. *Dis. Colon Rectum*, **2**, 465.

WORLD HEALTH ORGANISATION (1969). *Manual of the international statistical classification of diseases, injuries and causes of death*. 8th revision. WHO: Geneva.

WORLD HEALTH ORGANISATION (1976). *International classification of diseases for oncology*. WHO: Geneva.